# An analysis of the clinical features of children with early congenital Syphilis and Syphilitic Hepatitis

**DOI:** 10.1186/s12887-021-02932-5

**Published:** 2021-11-09

**Authors:** Hongling Yang, Huimin Zhang, Caiying Wang, Lin Pang

**Affiliations:** 1 Department of pediatrics,Beijing Ditan Hospital of China, Capital Medical University, 100015 Beijing, China; 2 Department of pediatrics, Beijing Ditan Hospital of China Capital Medical University, No. 8 of Jingshun East Street, Chaoyang District, 100015 Beijing, China

**Keywords:** Syphilitic hepatitis, Early congenital syphilis, Clinical features, Syphilis treatment, Penicillin

## Abstract

**Background:**

The infection rate of congenital syphilis is gradually increasing, the clinical manifestations of some children with congenital syphilis are abnormal liver function, which is given the clinical diagnosis of syphilitic hepatitis. At present, there are few studies on the clinical features of children with early congenital syphilis combined with syphilitic hepatitis, so we set out to do such a study. We compared the liver function indicators before and after the treatment of syphilis to find the clinical features that can provide guidance for clinical diagnosis and treatment.

**Methods:**

This study collected clinical data on 51 children with early congenital syphilis combined with syphilitic hepatitis in Beijing Ditan Hospital, affiliated with Capital Medical University, between April 2014 and October 2019. We observed their age, gender, clinical symptoms, and physical symptoms, as well as the pregnancy and childbirth history of their mothers. We also compared the liver function indicators before and after the treatment of the syphilis and analyzed the children’s clinical features.

**Results:**

The results of this study showed that the clinical manifestations in children with early congenital syphilis combined with syphilitic hepatitis were diverse. The most common clinical manifestation was anemia (56.9 %), followed by syphilitic rash (54.9 %), hands, feet, and whole-body peeling (35.3 %), and splenomegaly (29.4 %). Liver damage caused by a syphilis infection tends to result in elevated alanine aminotransferase, aspartate aminotransferase, and bilirubin, while albumin decreases. After the syphilis treatment, the liver function indexes were significantly improved compared with before treatment, and the difference was statistically significant (all p < 0.05).

**Conclusions:**

A child with abnormal liver function, especially with anemia, skin rash, peeling, abdominal distension, and hepatosplenomegaly should be highly suspected of having a syphilis infection. Once the diagnosis is made, the appropriate standard penicillin treatment should be started as soon as possible to improve the condition and prognosis of the child.

## Background

Congenital syphilis is a chronic systemic infectious disease caused by Treponema pallidum infecting the fetus through the placenta [1–3]. It can be divided into early congenital syphilis (0–2-year-old child) and late congenital syphilis (a child over two years). In China, the infection rate of congenital syphilis is gradually increasing, and the incidence of congenital syphilis in some areas has reached 1.03 % [4–6]. Treponema pallidum can invade all organs of the body and cause multiple organ damage. After infection, it can cause peripheral arteriovenous inflammation, intimates proliferation and thickening of vascular endothelium, and fibroblasts, which can lead to the thickening of vessel walls and stenosis and atresia of the vascular cavity [7]. Syphilitic hepatitis is liver damage caused by Treponema pallidum, which mostly occurs in late syphilis. The incidence of syphilitic hepatitis, liver damage, and cholestasis in early syphilis is relatively rare, with an incidence rate of 0.2–17.0 % [8]. Neonatal congenital syphilis can affect the skin, liver, kidneys, pancreas, spleen, lungs and intestines, as well as causing hematopoiesis outside of the bone marrow.

The key to the treatment of syphilitic hepatitis lies in the complete elimination of Treponema pallidum. At present, the treatment consists of administering penicillin, and the earlier the treatment begins, the better the results. The dosage should be sufficient and the course of treatment should be regular because irregular treatment can increase the risk of recurrence and promote the early occurrence of late syphilis damage [9].

Many children with early congenital syphilis have relatively hidden clinical manifestations, and abnormalities are only found during laboratory tests. Among the children with early congenital syphilis who were treated in the pediatric department of our hospital, some had abnormal liver function as the main manifestation, and the clinical diagnosis was syphilitic hepatitis.

At present, there are few studies on the clinical features of children with early congenital syphilis combined with syphilitic hepatitis. Therefore, we conducted this study to investigate the clinical features that can provide guidance for clinical diagnosis and treatment.

## 2 Materials and methods

**2.1 Research objec**t.

This study focused on children with early congenital syphilis combined with syphilitic hepatitis who were admitted to the Department of Pediatrics, Beijing Ditan Hospital Affiliated to Capital Medical University between April 2014 and October 2019. This study complies with the Helsinki Declaration of the World Medical Association and has been approved by the ethics committee of our hospital.

## 2.2 Inclusion and exclusion criteria

The inclusion criteria were as follows: (1) all selected cases met the diagnostic criteria of the 2010 Centers for Disease Control and Prevention diagnosis and treatment guidelines [10] and the diagnostic criteria for congenital syphilis issued by the Ministry of Health of CHINA [11] and were diagnosed as patients with early congenital syphilis combined with abnormal liver function and syphilitic hepatitis; The patients were diagnosed according to the clinical signs of illness, quantitative nontreponemal serum test (rapid plasma reagin [RPR]), examination of the cerebrospinal fluid (if their parents agreed) and also liver function tests. (2) the patient was 0–14 years old.

The exclusion criteria were as follows: (1) children with other infectious liver diseases, such as hepatitis A, B, C, D, E, or toxoplasmosis, other infections(varicella-zoster, parvovirus B19, rubella, cytomegalovirus, and herpes); (2) children with existing causes of abnormal liver function, such as drug-induced hepatitis, autoimmune liver disease, or inheritd metabolic disease; and (3) patients with incomplete case data.

## 2.3 Treatment

The dose of penicillin G was 50,000 U/kg every 12 h during the first seven days of life, and 50,000 U/kg every 8 h thereafter. The dose of penicillin G for older infants or children was 50,000 U/kg every 4–6 h, and the treatment time was 10–14 days. All the children were given a regular penicillin skin test before being given penicillin, and treatment could begin after the skin test was negative. Those with a positive penicillin skin test could have been given ceftriaxone 20–80 mg/kg once a day for 10–14 days as an alternative treatment. However, all the 51 patients included in this study were all treated with penicillin G, and reduced glutathione and compound glycyrrhizin and other drugs were used for liver protection treatment.

## 2.4 Main observation indicators

The main observation indicators of this study included the patient’s gender, age, onset time, hospitalization time, parental syphilis history, clinical manifestations, physical symptoms, laboratory findings, treatment, and prognosis. We also reviewed blood and urine routines, liver function, extremity bone films, chest radiographs, and other items, and we performed toluidine red unheated serum tests (TRUST), Treponema pallidum particle assays (TPPA), syphilis fluorescent antibody absorption tests (FTA-ABS-IgM), and liver and spleen ultrasounds and collected and analyzed data on various indicators of liver function before and after treatment.

## 2.5 Statistical methods

In this study, SPSS 22.0 software was used for statistical analysis. The measurement data of normal distribution was represented by mean ± standard deviation (x ± s) and the measurement data of non-normal distribution was represented by median and quartile. The count data were expressed as a percentage (%), normality was tested using the W-test, and the F-test was used to test the homogeneity of variance. The comparison between the two groups that obeyed the normal distribution was made using the t-test, the comparison between the groups that did not obey the normal distribution used the nonparametric test, and the chi-square test was used for count data. A p-value < 0.05 indicated that the difference was statistically significant.

## 3 Results

### 3.1 General information

A total of 51 children with a clear diagnosis of early congenital syphilis combined with syphilitic hepatitis were included in this study, comprising 32 males and 19 females. There were 14 premature babies and 37 full-term babies. There were 30 cases of normal delivery and 21 cases of cesarean section. The median age was 47 days (22, 72), and the onset time was two days to six months after birth.

## 3.2 Parents of children

Among the 51 children enrolled in this study, 41 had mothers with syphilis (with positive TRUST and TPPA), 7 had two parents with syphilis (with positive TRUST and TPPA), and 3 had parents with an unknown medical history. There were 35 cases whose mothers had not received regular syphilis treatment during pregnancy, 10 cases whose treatment was not standardized, 4 cases whose treatment was unknown, and 2 cases whose TRUST titer did not decrease after regular treatment.

## 3.3 The main signs and symptoms of the children

The results of this study showed that of the 51 children with early congenital syphilitic hepatitis, 28 cases presented with acral maculopapular rash, 18 cases with hand, foot, and whole-body peeling, and 15 cases with splenomegaly.

There were 12 cases of hepatomegaly, 14 cases of abdominal distension, 5 cases of diarrhea, 14 cases of vomiting, 29 cases of anemia, 4 cases of pneumonia, 11 cases of jaundice, 3 cases of hematuria, 7 cases of proteinuria, 11 cases of edema, 5 cases of fever, and 3 cases without signs and symptoms (see Table 1 for details).

**Table 1:** Symptoms and signs of 51 children with syphilis hepatitis

Symptoms and signs	Cases	Composition ratio%
anemia	29	56.9
rash	28	54.9
Peeling	18	35.3
Splenomegaly	15	29.4
vomit	14	27.5
abdominal distention	14	27.5
Hepatomegaly	12	23.5
edema	11	21.6
jaundice	11	21.6
proteinuria	7	13.7
diarrhea	5	9.8
fever	5	9.8
pneumonia	4	7.8
hematuria	3	5.9
No symptoms and signs	3	5.9

## 3.4 Syphilis-related antibodies and liver function test results

The results showed that TRUST and TPPA were both positive in the 51 children with syphilis.

Among them, 17 children had an FTA-ABS-IgM returned as positive. The TRUST titer of 30 children was 4 times or more that of their mother, of 6 children it was twice that of their mother, of 2 children it was the same as that of their mother, and of 5 children it was lower than that of their mother. The TRUST titer of the mothers of the remaining 8 children was unknown.

Before the treatment of the syphilis, the liver function test results showed that aspartate aminotransferase (AST) was elevated in 49 children (96.1 %), and the median AST was 123.7 (71.4, 175.9) U/L. A total of 40 children (78.4 %) with elevated alanine aminotransferase (ALT) had a median ALT of 86.9 (50.2, 170.5) U/L. A total of 20 children (39.2 %) with hypoalbuminemia had an average albumin value of 30.6 ± 6.4 g/L. There were 11 children (21.6 %) with elevated total bilirubin, the median of total bilirubin being 1.6 (0.5, 4.7) mg/dl, and a total of 9 children (17.6 %) had direct elevated bilirubin, the median direct bilirubin being 0.9 (0.3, 3.0) mg/dl.

After syphilis treatment, the median ALT was 56.8 (28.2, 94.5) U/L, the median AST was 67.0 (43.0, 122.6) U/L, the mean total protein was 59.9 ± 7.1 g/L, the mean albumin was 35.9 ± 4.3 g/L, the median total bilirubin was 0.5 (0.3, 2.9) mg/dl, and the median direct bilirubin was 0.4 (0.1, 1.6) mg/dl. The test results indicated that ALT, AST, total bilirubin, and direct bilirubin decreased and improved after treatment, while the test results of total protein and albumin improved compared with those before treatment. The above results were statistically significant before and after treatment (p < 0.05), but there was no significant difference in the examination results of glutamyl transpeptidase and alkaline phosphatase before and after treatment (p > 0.05) (see Table 2 for details).

**Table 2:** Liver function of children with syphilis hepatitis before and after treatment

Project (unit)	Before treatment	After treatment	statistic	*P*
AST(U/L)(IQR)	123.7(71.4,175.9)	67.0(43.0,122.6)	Z=-3.223	0.001*
TBIL(mg /dl)(IQR)	1.6(0.5, 4.7)	0.5(0.3, 2.9)	T=-4.548	0.000*
DBIL( mg /dl)(IQR)	0.9(0.3, 3.0)	0.4(0.1, 1.6)	T=-3.598	0.001*
ALP(U/L)(IQR)	315.4(221.3,440.4)	273.8(213.3,385.8)	Z=-1.205	0.228
GGT(U/L)(IQR)	170.6(82.2,275.7)	120.8(56.8,233.6)	Z=-1.670	0.095
TP(g/L)(mean ± SD )	56.3 ± 9.4	59.9 ± 7.1	T=-2.236	0.028*
ALB(g/L)(mean ± SD )	30.6 ± 6.4	35.9 ± 4.3	T=-4.891	0.000*
ALT(U/L)(IQR)	86.9 (50.2, 170.5)	56.8 (28.2, 94.5)	Z=-3.346	0.001*

ALT alanine aminotransferase, AST aspartate aminotransferase, TBIL total bilirubin, DBIL direct bilirubin, ALP alkaline phosphatase, GGT glutamyl transpeptidase, TP total protein, ALB albumin, IQR median was used, mean ± SD mean ± SD was used

## 3.5 Other laboratory test results

Before treatment, among the 51 children there were 29 (56.9 %) with reduced hemoglobin, 34 (66.7 %) with a white blood cell (WBC) count greater than 12 × 109 /L, 16 with thrombocytopenia (31.4 %), and 13 (24.5 %) with increased platelets. A total of 7 children (13.7 %) had positive urine protein, and 3 children (5.9 %) had hematuria.

## 3.6 Clinical prognosis and follow-up

The liver function of 46 children had significantly improved after two weeks of syphilis treatment. The liver function of 5 children did not improve significantly after the conventional liver protection treatment, and liver damage showed a trend of aggravation. However, the liver function gradually improved in 1–2 weeks after the addition of penicillin to treat the syphilis. There were 9 children with liver and spleen retraction during the follow-up 4–6 weeks after treatment.

## 4 Discussion

This study involved 51 children with a clear diagnosis of early congenital syphilis combined with syphilitic hepatitis. The results of the study showed that children with syphilitic hepatitis can have various clinical manifestations. The most common clinical manifestation was anemia, followed by syphilitic rash, hands, feet, and whole-body peeling, splenomegaly, vomiting, abdominal distension, and hepatomegaly. Syphilis treatment improved liver function indicators, especially alanine aminotransferase, aspartate aminotransferase, bilirubin, and albumin.

The specific causes of infantile hepatitis include infectivity, immunity, heredity, and metabolism, as well as poisons or drugs. Syphilis is an infectious cause of infantile hepatitis, accounting for about 1.2 % [12]. This study showed that 5.9 % of children with syphilitic hepatitis had no clinical signs or symptoms, while 27.5 % had vomiting and abdominal distension. The clinical manifestations of hepatitis caused by syphilis infection are diverse. If it is mild, patients may have no obvious signs or symptoms, but when it is severe, patients may have symptoms such as nausea, vomiting, or abdominal distension and often have clinical manifestations such as anemia, rash, peeling, yellowish skin, hepatosplenomegaly, and limb edema [13–15]. Some researchers have found that patients with severe syphilitic hepatitis may even have liver failure, decreased prothrombin activity (< 40 %) and total bilirubin greater than 170 umol/L. Common pathological manifestations of syphilitic hepatitis are hepatocyte necrosis, portal inflammatory infiltration, inflammation around the bile duct, and cholestasis [16–19]. The pathogenesis of syphilitic hepatitis has not been fully elucidated. Ridruejo et al. [20] found that liver biopsies of patients with syphilitic hepatitis had lymphocytic infiltration in the portal area, hepatocyte necrosis, noncaseating granuloma, and intrahepatic bile duct inflammation. Therefore, the underlying mechanism may be that the autoimmune response mediated by Treponema pallidum, or related immune complexes, directly damages the portal vein system, liver cells, and the intrahepatic bile duct system, leading to the onset of hepatitis.

Liver function examinations of the 51 children with syphilitic hepatitis before treatment indicated that liver damage caused by the syphilis infection was often due to the increase of ALT, AST, and bilirubin, and the decrease of proteins, especially albumin. After the diagnosis of syphilis, the children underwent standard treatment with penicillin, which in many cases had significant therapeutic effects, with rapid improvement in symptoms, and the obvious recovery of liver function, manifested by clear improvement in alanine aminotransferase, aspartate aminotransferase, albumin, and bilirubin.

Previous studies have shown that maternal serological titers are positively correlated with the risk of congenital syphilis [21]. In this study, 35 cases (68.6 %) of mothers of children diagnosed with early congenital syphilis were also found to have not received formal syphilis treatment during pregnancy. If pregnant women are not screened for syphilis before childbirth, and those with syphilis are not treated or do not undergo syphilis interruption treatment, the incidence of adverse pregnancy outcomes is as high as 69 %, and the proportion of children diagnosed with congenital syphilis is even higher [22, 23]. Therefore, screening for syphilis during pregnancy and early standardized treatment of infected pregnant women are of great importance in reducing the incidence of congenital syphilis.

In addition, children with abnormal liver function, especially those with anemia, skin rash, peeling, abdominal distension, and hepatosplenomegaly, should be highly suspected of having a syphilis infection, and standard appropriate penicillin treatment should be started as soon as possible after diagnosis to improve the child’s condition and prognosis.

This study has a number of shortcomings. First of all, it is a retrospective study, not a randomized controlled experiment, and there is no blind method, so there is still a certain risk of bias. Second, this study is a single-center clinical study, and a multi-center clinical study is still needed. Finally, the sample size is relatively small, so it is necessary to increase the sample size for further research.

## 5 Conclusions

Children with abnormal liver function, especially those with anemia, skin rash, peeling, abdominal distension, and hepatosplenomegaly, should be highly suspected of having a syphilis infection. Once the diagnosis is made, penicillin treatment should be started as soon as possible, which is essential to improve the condition and prognosis of the children.

## Data Availability

All data generated or analyzed during this study are included in this published article.

## Abbreviations

CDC: Centers for Disease Control and Prevention.
TPPA: treponema pallidum particle agglutination test.
TRUST: toluidine red unheated serum test.
FTA-ABS: fluorescent antibody adsorption test.
AST: aspartate aminotransferase.
ALT: alanine aminotransferase.
WBC: white blood cell.

## References

[1] Choudhri Y, Miller J, Sandhu J, Leon A, Aho J (2018). Infectious and congenital syphilis in Canada, 2010–2015. Can Commun Dis Rep.

[2] Brackett WJ, Standl TB (2019). Simulating Non-accidental Trauma with Worsening Findings:Congenital Syphilis. SN Comprehensive Clinical Medicine.

[3] Kanai M, Arima Y, Shimada T, Hori N, Yamagishi T, Sunagawa T, et al. Sociodemographic characteristics and clinical description of congenital syphilis patients and their mothers in Japan: a qualitative study, 2016. Sexual Health, 2018, 15.doi: 10.1071/SH18033. 10.1071/SH18033 30236211

[4] Hong FC, Liu JB, Feng TJ, Liu XL, Pan P, Zhou H (2010). Congenital Syphilis: An Economic Evaluation of a Prevention Program in China. Sexually Transmitted Diseases.

[5] Qin JB, Feng TJ, Yang TB, Hong FC, Lan LN, Zhang CL (2014). Risk factors for congenital syphilis and adverse pregnancy outcomes in offspring of women with syphilis in Shenzhen, China: a prospective nested case-control study. Sexually Transmitted Diseases.

[6] Zhang X, Yu Y, Yang H, Xu HY, Vermund SH, Liu K (2018). Surveillance of Maternal Syphilis in China: Pregnancy Outcomes and Determinants of Congenital Syphilis. Medical ence Monitor.

[7] Hu Yamei and Jiang Zaifang, Zhu Futang Practical Pediatrics (8th edition) People’s Medical Publishing House, 2015

[8] Noto P, Del Nonno F, Licci S (2008). Early syphilitic hepatitis in an immunocompetent patient: really so uncommon? Int. J STD AIDS.

[9] The STD Control Center of the Chinese Center for Disease Control and Prevention, the Venereology Group of the Dermatology and Venereology Branch of the Chinese Medical Association, the STD Sub-specialty Committee of the Dermatologist Branch of the Chinese Medical Doctor Association (2020). Guidelines for diagnosis and treatment of syphilis, gonorrhea and genital tract Chlamydia trachomatis infection (2020). Chinese Journal of Dermatology.

[10] Workowski KA, Berman S, Centers for Disease Control and Prevention(CDC). Sexually transmitted diseases treatment guidelines, 2010.MMWR Recomm Rep,2010,17,59(RR-12):1–110. 21160459

[11] Ministry of Health of the People’s Republic of China (2007). Syphilis diagnostic criteria(WS268-2007)[S].

[12] Yolanda CC, Cristian VR (2013). Anicteric Hepatitis as Congenital Syphilis Manifestation. Infectio.

[13] Zoufaly A, Onyoh EF, Tih PM, Awasom CN, Feldt T (2012). High prevalence of hepatitis B and syphilis co-infections among HIV patients initiating antiretroviral therapy in the north-west region of Cameroon. International Journal of Std & Aids.

[14] Demirel Y, Duran B, Toktamis A, Erden O, Cetin M (2004). Seroprevalence of syphilis, hepatitis B and C, and human immunodeficiency virus infections among women. Saudi Medical Journal.

[15] Schuelter-Trevisol F, Custodio G, Silva A C B D (2013). HIV, hepatitis B and C, and syphilis prevalence and coinfection among sex workers in Southern Brazil. Revista da Sociedade Brasilra de Medicina Tropical.

[16] Crum-Cianflone N, Weekes J, Bavaro M (2009). Syphilitic hepatitis among HIV-infected patients. International Journal of Std & Aids.

[17] Lo JO, Harrison RA, Hunter AJ. Syphilitic hepatitis resulting in fulminant hepatic failure requiring liver transplantation.Journal of Infection,2007,54(3):e115-e117. doi: 10.1016/j.jinf.2006.07.005. 10.1016/j.jinf.2006.07.005 16939692

[18] Mullick CJ, Liappis AP, Benator DA, Roberts AD, Parenti DM, Simon GL. Syphilitic Hepatitis in HIV-Infected Patients: A Report of 7 Cases and Review of the Literature. Clinical Infectious Diseases(10):1561–1561.doi: 10.1086/425501. 10.1086/425501 15546070

[19] Noto P, Nonno FD, Licci S, Chinello P, Petrosill N (2008). Early syphilitic hepatitis in an immunocompetent patient: really so uncommon?. International Journal of Std & Aids.

[20] Ridruejo E,Mordoh A,Herrera F,Avagnina A,Mando OO.Case report: severe cholestatic hepatitis as the first symptom of secondary syphilis.Dig Dis Sci,2004,49(9):1401.doi:10.1023/b:ddas.0000042237.40205.c6. 10.1023/b:ddas.0000042237.40205.c6 15481310

[21] Dou LX, Wang AL, Wang XY, Wang Q, Qiao YP, Su M,et al. Analysis of factors influencing the treatment of pregnant women with syphilis and their adverse pregnancy outcomes.Chinese Journal of Obstetrics and Gynecology, 2016,51(7):538-541.10.3760/cma.j.issn.0529-567X.2016.07.011

[22] Hawkes S,Matin N,Broutet N, Low N.Effectiveness of interventions toimprove screening for syphilis in pregnancy: a systematic review and meta-analysis.Lancet Infect Dis,2011,11(9): 684–691.doi:10.1016/S1473-3099(11)70104-9. 10.1016/S1473-3099(11)70104-9 21683653

[23] O’Connor NP, Gonzalez BE, Esper FP, Tamburro J, Kadkhoda K, Foster CB. Congenital syphilis:Missed opportunities and the case for rescreening during pregnancy and at delivery. IDCases, 2020, 22.doi:10.1016/j.idcr.2020.e00964. 10.1016/j.idcr.2020.e00964 PMC7528198 33024697

